# Hospital Human Resource Managers’ Perspectives on Organizational Readiness for Generative AI Skills: Qualitative Descriptive Study

**DOI:** 10.2196/74339

**Published:** 2026-09-17

**Authors:** Zhuo Gao, Deliang Liu, Yanxia He, Yan Zhao, Yan Zuo, Jianjun Zhang, Jingjing Zheng

**Affiliations:** 1 Department of Human Resource Management, Beijing Geriatric Hospital, Bejing, China; 2 Audit Office, Beijing Geriatric Hospital, Bejing, China; 3 General Office, Beijing Geriatric Hospital, Beijing, China; 4 West China School of Nursing, Sichuan University, Sichuan, China/Department of Gynecology and Obstetrics Nursing, West China Second University Hospital, Sichuan University/Key Laboratory of Birth Defects and Related Disease of Women and Children (Sichuan, Chengdu, China; 5 Department of Gynecology and Obstetrics, West China Second University Hospital, Sichuan University/Key Laboratory of Birth Defects and Related Diseases of Women and Children (Sichuan University), #20 3rd Section, Renmin Nan Road, Chengdu, China, 86 13348886426; 6 Beijing Geriatric Hospital, 118 Wenquan Road, Haidian District, Beijing, 100000, China

**Keywords:** generative AI, large language models, hospital administration, human resource management, organizational readiness, workforce development, qualitative research, thematic analysis, health informatics, implementation

## Abstract

**Background:**

Generative AI (GenAI) is increasingly entering health care through documentation support, communication tools, educational content generation, and other knowledge-intensive functions. However, organizational adoption remains uneven, and concerns related to privacy, security, output reliability, governance, workflow fit, and infrastructure continue to limit broader implementation. Although the literature increasingly discusses the skills health care workers may need in the GenAI era, less is known about how hospital managers view the organizational readiness required before such skills can be expected across the workforce. Hospital human resource (HR) managers are especially important in this regard because they are involved in training, competency development, workforce planning, and organizational change.

**Objective:**

This study aimed to explore how hospital HR managers perceived the relevance of GenAI-related skills, the organizational barriers to broader implementation, and the forms of preparation they considered realistic in the current stage of adoption.

**Methods:**

We conducted a descriptive qualitative study in 2 tertiary hospitals in China—1 in Beijing and 1 in Sichuan Province. Purposive sampling was used to recruit HR managers involved in staffing, training, competency development, or workforce planning. Semistructured telephone interviews were conducted between January 15 and February 9, 2025. Interviews were audio-recorded, transcribed verbatim, and analyzed using inductive thematic analysis. The researchers familiarized themselves with the transcripts, generated initial codes, grouped related codes into categories, and developed themes through iterative comparison, team discussion, and refinement. Rigor was supported through reflexive memoing, audit trail documentation, team debriefing, and participant validation.

**Results:**

Fifteen HR managers participated. Participants did not describe mature or formalized GenAI competency systems within their institutions. Instead, they described an early-stage organizational environment characterized by strategic recognition of GenAI, informal and uneven experimentation, uncertain governance, and substantial variation in readiness across roles and settings. Three themes were identified: (1) GenAI was viewed as increasingly inevitable and relevant to hospital work, but not yet as a stable universal competency requirement; (2) organizational unreadiness, including infrastructure limitations, lack of approved systems, unclear policy boundaries, and workforce heterogeneity, constrained any move toward universal mandates; and (3) participants preferred phased, role-specific preparation over immediate compulsory standards. They emphasized that readiness depended on secure access, governance, workflow alignment, and differentiated training pathways rather than broad top-down requirements.

**Conclusions:**

Hospital HR managers perceive GenAI as increasingly important but not yet governable through universal competency expectations. Their accounts suggest that hospitals are not simply deciding whether to adopt GenAI, but are negotiating when, for whom, and under what safeguards GenAI-related skills become legitimate workforce expectations. Readiness-based, phased, and role-specific approaches may therefore be more appropriate than premature competency mandates in the early stages of hospital GenAI implementation.

## Introduction

AI is increasingly embedded across health care delivery, administration, education, public health, and research [1,2]. The current wave of generative AI (GenAI) raises a distinct set of organizational and workforce questions because, unlike many earlier AI systems that primarily classify, predict, or detect, GenAI systems generate new text, summaries, recommendations, and conversational outputs through prompt-based interaction [3-5]. In health care, GenAI is already being explored for documentation, patient communication, telehealth interaction, educational drafting, literature summarization, coding support, and other knowledge-intensive tasks [3,6].

This distinction matters because the practical demands and risks associated with GenAI are not identical to those associated with predictive, discriminative, or diagnostic AI [5]. GenAI systems can produce plausible but inaccurate outputs, vary according to prompt quality, obscure the provenance of generated content, and create privacy and security risks when users enter sensitive information into external systems [3,5]. Recent reporting guidance for chatbot health advice studies has likewise emphasized prompt engineering, query strategy, evaluation, and transparency as specific methodological concerns for GenAI-based systems [7,8].

The literature on AI capability in health care has made important progress, especially in identifying foundational domains such as AI literacy, data literacy, ethics, communication, and critical appraisal of AI-supported outputs [9,10]. At the same time, more recent GenAI-focused work argues that health care workers increasingly need practical abilities related to prompting, verification of generated content, recognition of hallucinated or low-reliability outputs, and appropriate escalation to human oversight [5,9]. Educational commentaries in medicine have similarly argued that the rapid spread of AI, particularly GenAI, requires targeted training rather than generic digital upskilling alone [11,12].

However, much of the current literature is normative [9,10]. It is often stronger on what clinicians or staff should know than on how health care organizations perceive the feasibility of expecting such skills across a heterogeneous workforce [10,13]. Evidence from systematic reviews shows that health care professionals’ experiences with AI are mixed and are shaped by variability in understanding, trust, perceived usefulness, and workflow consequences [13-15]. Reviews of professionals’ concerns likewise show persistent anxiety about safety, accountability, deskilling, trust, and professional displacement [14-16].

This gap is especially important in hospitals, where AI adoption is shaped not only by tool performance but also by infrastructure, governance, workflow fit, leadership, data access, and implementation capacity [17-19]. A recent survey of US health systems found active AI adoption during the early GenAI era, but also substantial barriers related to implementation, resources, and organizational readiness [20]. Broader institutional frameworks in health care similarly emphasize that AI adoption depends on multiple dimensions, including technology, human factors, organizational structures, and external context [19,21]. Work on learning health systems further suggests that hospitals need staged implementation strategies rather than one-step deployment [18,22].

GenAI also reaches hospitals through a different pathway than many prior clinical AI tools. Publicly available chatbots and content-generation systems are easy to access and experiment with, which means use may begin informally before institutional policy and secure infrastructure mature [3,6,23]. That sequence has intensified concern about data privacy, regulatory compliance, output reliability, and governance [5,23]. Recent health policy commentaries have therefore stressed the need for transparency, guardrails, and responsible regulation for AI in health care [2,24,25].

The organizational implications of GenAI are not limited to clinicians. Hospital human resource (HR) managers are centrally involved in recruitment, competency discussions, training, staff development, job redesign, and organizational change. That makes HR a key intermediary between technological ambition and workforce feasibility. This inference is consistent with the broader HR literature, which increasingly frames AI and algorithmic technologies as reshaping workforce systems, capability development, governance, and employee relations [26-28]. Recent work in HR and management also highlights the need to address bias, fairness, and governance when AI is used in HR-related decision processes [29-31]. Research on GenAI in HR management (HRM) similarly emphasizes both transformative promise and integrated risk [32-34].

This perspective is especially relevant in health care, where HR functions must operate under tight constraints related to professional roles, patient safety, training equity, and organizational accountability. AI-related change in hospital settings is therefore not simply a matter of introducing new tools. It is also a matter of deciding when skill expectations become legitimate, how those expectations differ by role, and what institutional supports are needed before broader requirements can be considered. The current implementation literature supports such a view by showing that adoption depends on readiness, leadership, sociotechnical fit, and trust rather than on technical performance alone [13,18,35,36].

Despite rapid growth in the AI and GenAI literature, 3 gaps remain. First, most health care AI work focuses on clinicians, educators, technical systems, or patient-facing use cases, while HR managers remain underexamined [3,10,13]. Second, discussions of AI capability in hospitals often conflate GenAI with broader AI categories, even though prompt-based content generation creates distinct practical, governance, and competency issues [4,5,8]. Third, there is limited empirical evidence on how hospital managers interpret the organizational conditions under which GenAI-related skill expectations become realistic, appropriate, or premature [17,19,20].

The Chinese hospital context makes this question especially timely. China is rapidly expanding AI use in health care while simultaneously confronting regulatory, infrastructural, and evaluative challenges [37-39]. Chinese regulatory analyses have noted the growing importance of governance, privacy, software regulation, and evaluation consistency for AI-enabled health technologies [38-40]. At the same time, hospitals may vary substantially in digital infrastructure, local policy maturity, and organizational capacity to support GenAI safely and equitably. That makes hospital managers’ perspectives particularly valuable in understanding early adoption conditions [21,37].

The present study therefore explores how hospital HR managers in 2 Chinese tertiary hospitals perceive the emerging expectation that hospital staff should develop GenAI-related skills. Rather than assuming the existence of a mature competency framework, the study examines how managers conceptualize GenAI’s relevance, what organizational barriers they perceive to broader implementation, and what forms of preparation they consider realistic at the current stage. When participants refer to imaging, predictive, or diagnostic AI, those references are treated analytically as evidence of prior AI exposure and organizational digital baseline rather than as direct evidence of GenAI use. This distinction is consistent with current literature emphasizing that GenAI should not be collapsed into AI as a single undifferentiated category [4,5,19].

The study addresses 3 questions: how HR managers understand the relevance of GenAI-related skills in hospital work, what infrastructural, governance, and workforce barriers they perceive in moving toward broader adoption, and what phased or role-specific forms of preparation they consider appropriate. By focusing on HR managers as organizational sense-makers, the study contributes to a more realistic account of hospital GenAI adoption during a period when use may be emerging, uneven, and only partially governed.

## Methods

### Study Design

This study used a descriptive qualitative design to examine how hospital HR managers understood the emerging expectation that hospital staff should develop skills related to GenAI. The study focused on participants’ practice-based perspectives on preparedness, implementation barriers, and the conditions under which GenAI-related skill expectations might become realistic in hospital settings.

A qualitative descriptive approach was selected because the research question focused on how HR managers understood and described an emerging organizational issue in practical, applied terms rather than on testing predefined categories. The analytic aim was to provide a clear, practice-oriented description of participants’ accounts while remaining close to their language and practical concerns. Although the interview guide explicitly asked about GenAI-related skills, some participants also referred to other AI applications already present in hospitals, such as imaging, predictive, or decision-support systems. In the analysis, such references were treated as descriptions of participants’ prior AI exposure and organizational digital baseline, rather than as direct evidence of GenAI use.

### Study Setting and Sampling

The study was conducted in 2 tertiary hospitals in China, 1 located in Beijing and 1 in Sichuan Province, between January 15 and February 9, 2025. The hospitals were selected purposively to capture HR perspectives from 2 large institutions operating in different regional and organizational contexts and at different stages of digital development. The purpose of including both sites was to broaden contextual variation, not to establish a formal comparative design.

Purposive sampling was used to recruit HR managers who were likely to have direct experience with workforce planning, competency discussions, or digital training decisions. Participants were eligible if they met all of the following criteria: they had at least 1 year of hospital HR management experience, were currently involved in staffing, training, competency development, or workforce planning, and supervised or supported clinical staff, administrative staff, or both. Interim appointees and HR personnel whose roles were restricted to routine administrative processing without involvement in workforce capability planning were excluded.

### Recruitment and Participants

Eligible participants were identified through departmental rosters and liaison contacts at each hospital. Invitation messages were circulated locally, and interested individuals were contacted by the research team. Seventeen HR managers were approached, and 15 agreed to participate, yielding a participation rate of 88.2%.

### Data Collection

Data were collected through semistructured telephone interviews. Telephone interviews were chosen because they allowed timely access to managerial participants across 2 sites while minimizing disruption to hospital operations. This format also increased scheduling flexibility for participants with limited availability. A limitation of telephone interviewing is the reduced access to visual nonverbal cues. To address this, interviewers recorded contemporaneous field notes on relevant paralinguistic features, including pauses, hesitations, changes in tone, emphasis, and laughter.

The interview guide was developed through team discussion based on the study aim and relevant literature on GenAI in health care, workforce capability development, and digital implementation in organizations. The guide was pilot-tested with 2 HR managers who were not included in the final sample. Minor revisions were made to improve clarity and sequencing. The guide included questions on 4 domains: participants’ understanding of GenAI and its relevance to hospital work, perceptions of whether GenAI-related skills should be expected of staff, perceived barriers and risks, and recommendations for implementation, training, and governance. The full interview guide is provided as Multimedia Appendix 1.

All interviews were conducted in Chinese by trained qualitative researchers. Interviews were audio-recorded with permission and transcribed verbatim within 48 hours.

Because the manuscript was prepared in English, quotations were translated from Chinese into English after transcription. To preserve readability, translated quotations were lightly edited for grammar and syntax without changing substantive meaning. This was done to reduce distraction from literal translation artifacts. Because AI-assisted translation support was used during manuscript preparation, some nuance related to emotional tone, colloquial expression, or regional language use may have been attenuated.

### Data Analysis

Data collection and analysis proceeded concurrently. Transcripts were analyzed using inductive thematic analysis within the qualitative descriptive design to identify patterned meanings related to perceptions of GenAI relevance, organizational barriers, and preferred forms of workforce preparation.

The analytic process involved several stages. First, the researchers read each transcript repeatedly and reviewed the audio recordings as needed to achieve familiarity with the data. Reflexive notes were made during this stage to document and critically examine the research team’s prior assumptions about AI modernization and workforce preparedness. In particular, the researchers recognized 3 preunderstandings that could shape interpretation: first, an initial expectation that GenAI would be described as an increasingly necessary future skill in hospital work; second, a concern that hospital adoption would be constrained by governance, privacy, infrastructure, and implementation capacity; and third, a tendency to view managerial discussion of AI positively as evidence of modernization rather than as potentially tentative, ambiguous, or contested. These assumptions were discussed during analysis to reduce the risk of overinterpreting strategic interest in GenAI as evidence of organizational readiness or mature implementation.

Second, segments of text relevant to the research question were identified and coded. Third, initial codes were developed to capture the substantive content of participants’ accounts. During coding, the team explicitly distinguished between material concerning GenAI-related practices, such as prompting, generating text, checking outputs, or drafting assistance, and material concerning non-GenAI systems, such as diagnostic imaging or predictive analytics. Material in the latter category was retained only when it clarified how prior AI exposure shaped participants’ understanding of GenAI readiness.

Fourth, related codes were grouped into preliminary categories. Fifth, categories were iteratively compared within and across transcripts and consolidated into subthemes and themes through team discussion and constant comparison. Sixth, the themes were refined into an analytic account of participants’ perspectives. Seventh, the thematic structure was refined through team debriefing and participant validation.

Two researchers independently coded an initial subset of transcripts and compared their coding to develop a shared coding framework. Differences were discussed until consensus was reached. The remaining transcripts were then coded using the agreed framework, while still allowing new codes and refinements to emerge. An audit trail documented code development, analytic memos, interpretive decisions, and theme revisions. An analytic summary showing the progression from research questions and corresponding interview questions to illustrative codes, subcategories, and final themes is presented in Table 1.

Concurrent analysis suggested that later interviews largely confirmed the existing thematic structure without substantially altering the developing interpretation. Although new nuances continued to emerge, no major thematic shifts were observed in the final interviews.

**Table 1.** Analytic progression from research questions and corresponding semistructured interview questions to illustrative codes, subcategories, and final themes in a descriptive qualitative study of hospital human resource managers at 2 tertiary hospitals in Beijing and Sichuan, China, January 15 to February 9, 2025^a^.

Research question	Corresponding interview questions from Multimedia Appendix 1	Illustrative early codes	Subcategories	Final themes
How do hospital HR^b^ managers understand the relevance of GenAI^c^-related skills in hospital work?	Q3. In your hospital, who makes decisions about adopting GenAI-related tools, and how is HR involved? Q4. What GenAI-related use is currently happening in your hospital (formal programs, informal use, or none)? Q5. When you hear “GenAI competency,” what does it mean in your hospital context? Q6. Which GenAI competencies should be expected of all staff, if any, and which should be role-specific? Q8. What benefits do you anticipate from developing GenAI competency?	“AI is inevitable”; “already being used informally”; “future of work”; “documentation and communication support”; “HR needs to think ahead”; “not yet mainstream”; “competency expectations still unclear”	Strategic recognition of GenAI; informal experimentation and early use; anticipatory preparation; unsettled expectations about competency	GenAI was viewed as inevitable, but not yet a stable competency requirement
What infrastructural, governance, and workforce barriers do HR managers perceive in moving toward broader adoption of GenAI-related skills?	Q7. Do you think GenAI competency should become a formal, required competency standard in hospitals? Why or why not? Q9. What risks or concerns do you see? Q10. What barriers would make a competency requirement difficult to implement in your hospital? Q11. How does readiness vary across roles or cohorts, and why? Q15. What governance and participation are needed to implement this responsibly?	“old hardware”; “no approved systems”; “unclear rules”; “privacy risk”; “legal responsibility unclear”; “uneven confidence”; “different roles need different things”; “younger staff more receptive”; “senior staff hesitant”; “relevance varies by role”	Infrastructure and system constraints; governance and accountability gaps; privacy and safety concerns; uneven readiness across roles and career stages	Organizational unreadiness limited any move toward universal mandates
What forms of preparation do HR managers consider realistic and appropriate at the current stage of GenAI adoption?	Q6. Which GenAI competencies should be expected of all staff, if any, and which should be role-specific? Q12. If you were designing a competency pathway, what tiers or stages would you propose for different roles? Q13. How should GenAI competency be taught and supported in practice? Q14. How should competency be assessed and maintained over time, and what would “minimum acceptable competency” look like? Q15. What governance and participation are needed to implement this responsibly, and what are your top recommendations for the next 3 to 6 months?	“tiered training”; “role-specific learning”; “basic versus advanced skills”; “prompting and checking outputs”; “human review needed”; “minimum safe-use capability”; “workflow fit matters”; “frontline staff should shape workflows”; “phased implementation”	Tiered competency pathways; practical and bounded skill expectations; workflow-aligned implementation; collaborative design and governance	Participants preferred phased, role-specific preparation over immediate compulsory standards

a Codes are illustrative examples rather than an exhaustive codebook.

b HR: human resource.

c GenAI: generative AI.

### Rigor and Trustworthiness

Credibility was supported through repeated reading of transcripts, concurrent analysis during data collection, member checking with selected participants, and team debriefing. Dependability was strengthened through an audit trail documenting interview procedures, coding decisions, and theme development. Confirmability was enhanced through reflexive memo writing and team discussion regarding the research team’s prior assumptions about AI in hospitals. Before and during analysis, the team explicitly acknowledged that they were inclined to view AI as an important direction of hospital development, to expect uneven organizational readiness across roles and settings, and to interpret references to AI use as signals of institutional progress. These assumptions were revisited throughout coding and theme development so that participant accounts emphasizing uncertainty, limited use, governance gaps, or skepticism were retained as central findings rather than being subordinated to a modernization narrative. Transferability was supported by providing contextual detail about the participating hospitals, participant roles, and the early-stage implementation environment in which GenAI was being discussed.

### Ethical Considerations

The study was exempt from formal ethical review as determined by the Ethics Committee of Beijing Geriatric Hospital and reciprocally recognized by the participating hospital in Sichuan. The exemption was based on the applicable institutional academic ethics policies because the study involved staff interviews about professional practice and did not involve patients, clinical interventions, or access to sensitive clinical data. No formal waiver or exemption protocol number was issued. All participants provided verbal informed consent before the interview and were informed of the study purpose, voluntary nature of participation, confidentiality safeguards, and their right to decline any question or withdraw at any time.

Participants were anonymized using alphanumeric identifiers indicating site and participant number, such as BJ-HR04 and SC-HR03. Audio files, transcripts, and demographic data were stored on password-protected devices accessible only to the research team.

## Results

### Participant Characteristics

Fifteen HR managers participated in the study, including 8 from the Beijing hospital and 7 from the Sichuan hospital. Participants ranged from 34 to 52 years of age, with a mean age of 42.3 (SD 5.2) years. The sample included 10 women and 5 men. Participants came from diverse professional backgrounds, including health care administration, HR management, and clinical disciplines before transition into HR-related roles. Interviews lasted 25 to 49 minutes, with a mean duration of 34 (SD 6.8) minutes.

Across both sites, participants described GenAI as visible and increasingly relevant, but not yet embedded within formal hospital-wide standards. Their accounts did not indicate the existence of mature GenAI competency systems. Instead, they described an environment characterized by informal experimentation, unequal exposure, uncertain governance, and uneven infrastructure. Participant characteristics are summarized in Table 2.

**Table 2.** Characteristics of hospital human resource managers interviewed in a descriptive qualitative study of organizational readiness for generative AI skills at 2 tertiary hospitals in Beijing and Sichuan, China, January to February 2025 (N=15)^a^.

Characteristic	Value
Site, n	
Beijing	8
Sichuan	7
Sex, n	
Female	10
Male	5
Age (y)	
Range	34‐52
Mean (SD)	42.3 (5.2)
Years in HR^b^	
Range	1‐23
Mean (SD)	10 (6.1)
Role, n	
HR director/deputy director	5
Recruitment/staffing manager	14
Training and development manager	12
Performance/assessment manager	10
Workforce planning/organizational development manager	8
Comprehensive/general HR administration	13
Professional background, n	
Health care administration	6
Human resource management	5
Clinical field before HR role	4
Interview length, min	
Range	25‐49
Mean (SD)	34 (6.8)

a Role categories were not mutually exclusive; participants could hold multiple role responsibilities concurrently.

b HR: human resource.

### Overview of Themes

**Figure 1.**
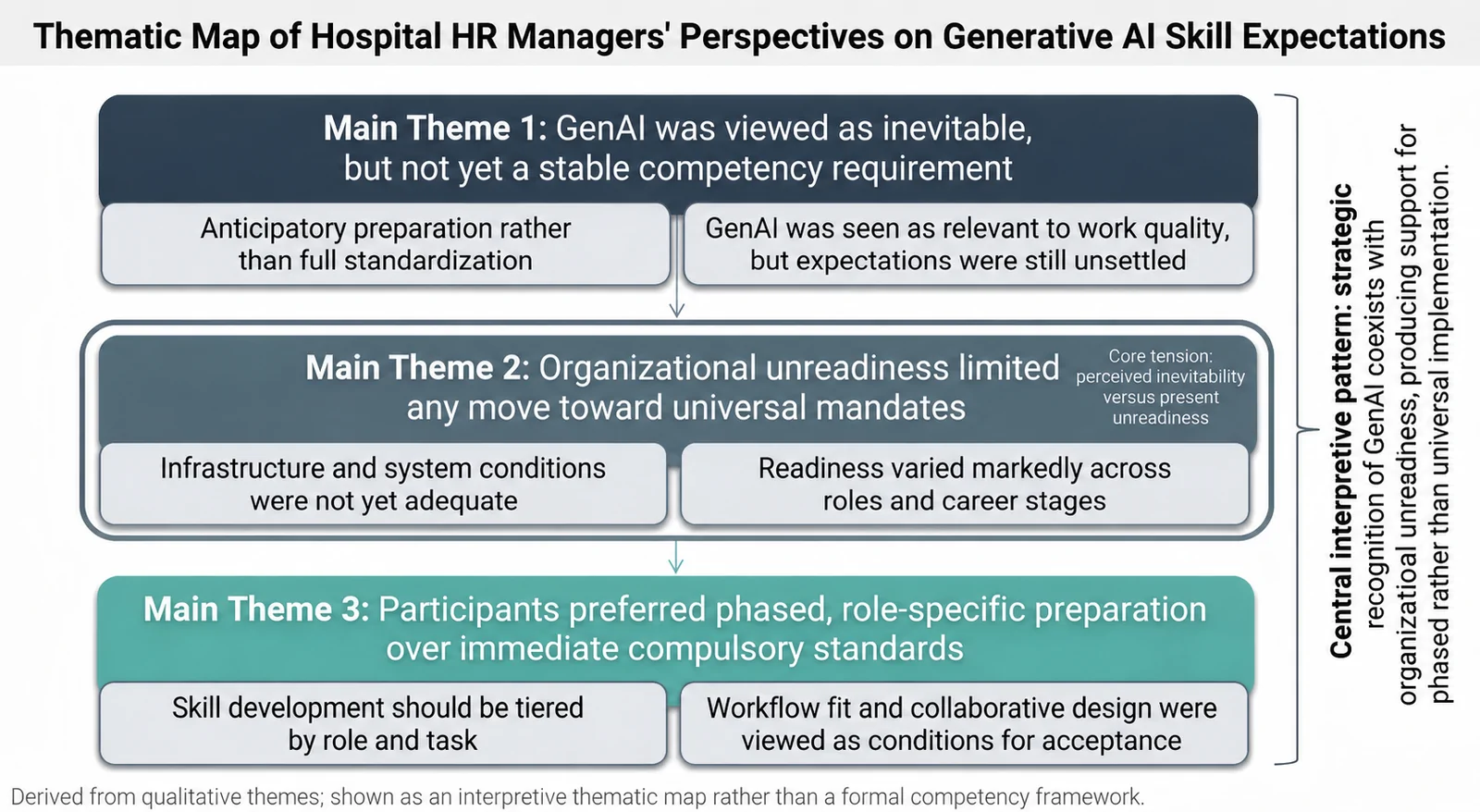
Thematic map of hospital human resource (HR) managers’ perspectives on generative AI (genAI) skill expectations, derived from semistructured interviews in a descriptive qualitative study at 2 tertiary hospitals in Beijing and Sichuan, China, January 15 to February 9, 2025. The map shows the relationship among perceived inevitability, organizational unreadiness, and preference for phased, role-specific preparation; it is an interpretive thematic summary rather than a validated competency framework.

Three themes were identified: first, GenAI was viewed as inevitable, but not yet a stable competency requirement; second, organizational unreadiness limited any move toward universal mandates; and third, participants preferred phased, role-specific preparation over immediate compulsory standards. The relationship among the 3 themes is summarized in Figure 1.

Table 3 summarizes the qualitative themes and subthemes.

**Table 3.** Themes and subthemes derived from semistructured interviews with hospital human resource managers in a descriptive qualitative study of organizational readiness for generative AI skills at 2 tertiary hospitals in Beijing and Sichuan, China, January to February 2025^a^.

Theme	Subtheme	Central interpretive meaning
GenAI^b^ was viewed as inevitable, but not yet a stable competency requirement	Anticipatory preparation rather than full standardization GenAI was seen as relevant to work quality, but expectations were still unsettled	Participants saw GenAI as strategically unavoidable, but not yet ready for universal formal requirements Participants linked GenAI to future improvements, yet described skill expectations as emergent and unclear
Organizational unreadiness limited any move toward universal mandates	Infrastructure and system conditions were not yet adequate Readiness varied markedly across roles and career stages	Material and system constraints made broad skill mandates premature Participants described large differences in relevance, motivation, and digital confidence
Participants preferred phased, role-specific preparation over immediate compulsory standards	Skill development should be tiered by role and task Workflow fit and collaborative design were viewed as conditions for acceptance	Participants favored bounded, practical skill pathways rather than uniform requirements Staff involvement and workflow alignment were seen as necessary for sustainable implementation

a The table summarizes the final thematic structure.

b GenAI: generative AI.

### Theme 1: GenAI Was Viewed as Inevitable, but Not Yet a Stable Competency Requirement

#### Overview

Participants consistently described GenAI as an emerging part of hospital work that HR departments could not ignore. They associated it with future changes in documentation, communication, administrative support, patient education, and knowledge work more broadly. However, they did not describe a settled institutional view that all hospital staff should already be held to a common GenAI competency standard. One participant summarized this forward-looking but unsettled position:

*AI is inevitable. Staff are already trying AI applications, but mostly in a random way. There may be efficiency gains, but it is not yet mainstream. As HR managers, we need to think about it in advance*.

[BJ-HR04]

This kind of account positioned GenAI as strategically important but operationally immature. Participants were not arguing against skill development. Rather, they were saying that a future-oriented issue had arrived before institutional rules, pathways, and boundaries had fully formed.

#### Subtheme 1.1: Anticipatory Preparation Rather Than Full Standardization

Participants often emphasized the need to begin preparing staff before GenAI tools became normalized in routine work. Their emphasis, however, was on anticipatory preparation rather than full standardization. They described the current period as one in which hospitals should begin building awareness and safe-use capacity, not one in which they should impose rigid organization-wide requirements. One participant stated:

*If we wait until the tools become mainstream, it will be too late. People need some preparation before they are suddenly expected to use them in daily work*.

[BJ-HR04]

In several interviews, participants explained GenAI through comparisons with earlier forms of hospital AI. When they referred to diagnostic imaging systems, predictive tools, or other AI-enabled platforms, they appeared to be drawing on prior institutional experience to make sense of GenAI preparedness. In the present analysis, such statements were interpreted as evidence of an organizational AI baseline rather than as evidence of GenAI itself:

*Some staff in the HR department and hospital office are already using AI to draft documents and summarize materials. It is fast, but it also carries risks. AI-generated content can still be wrong, and it may not fit our actual local situation*.

[BJ-HR06]

#### Subtheme 1.2: GenAI Was Seen as Relevant to Work Quality, but Expectations Were Still Unsettled

Participants frequently linked GenAI to possible gains in speed, documentation quality, communication support, and information organization. Even so, they also described a lack of clarity regarding what should count as acceptable or necessary skill.

This unsettledness appeared in two forms. First, participants were unsure how far GenAI-related expectations should extend across different staff groups. Second, they described a gap between strategic enthusiasm and operational specification. In other words, GenAI was already being treated as consequential, but the expected level of competence remained undefined:

*It is not enough to teach them how to ask questions or generate content. They also need to understand where AI is likely to fail. They need a kind of reflex so that when they see an AI output, they can immediately judge which parts require human review. You cannot simply trust the machine*.

[SC-HR02]

### Theme 2: Organizational Unreadiness Limited Any Move Toward Universal Mandates

#### Overview

The second theme captured participants’ view that hospitals were not yet ready to make GenAI-related skills a universal requirement. Their accounts pointed to unreadiness at multiple levels, including infrastructure, governance, workflow integration, and workforce heterogeneity. They did not reject GenAI as a strategic direction. Rather, they questioned whether the organizational conditions necessary for fair and meaningful implementation were currently in place.

#### Subtheme 2.1: Infrastructure and System Conditions Were Not Yet Adequate

Participants repeatedly described a gap between strategic discourse about AI and the practical conditions of daily work. Material limitations were especially salient. Participants referred to outdated computers, uneven system access, lack of approved tools, and broader digital infrastructure constraints. One participant remarked:

*We are being asked to introduce AI-based documentation, but many nurses are still using computers that are more than 10 years old. Requiring skills before the infrastructure is ready is like teaching swimming without water*.

[SC-HR05]

This quotation captured more than a complaint about equipment. It reflected a broader interpretive position that skill expectations and infrastructure readiness must develop together. In participants’ accounts, asking staff to demonstrate capability in the absence of usable systems would produce frustration rather than meaningful adoption.

Another aspect of unreadiness concerned governance. Some participants described a situation in which staff were already experimenting informally with publicly available AI tools, while hospitals had not yet established clear rules on approved use, data entry boundaries, or verification responsibilities. A participant from Sichuan elaborated:

*In fact, people at the working level are already using it. Many are using tools like Kimi to draft materials, including some clinical content such as patient education materials and even some case-related documents. But the hospital still has not issued clear guidance. We know patient data definitely cannot just be pasted into it, but what other content is acceptable? And if the generated content creates legal risk, is the user responsible or the department? At the moment, all of that is still a blank area*.

[SC-HR01]

#### Subtheme 2.2: Readiness Varied Markedly Across Roles and Career Stages

Participants described substantial differences in readiness across staff groups. They did not view the workforce as a single population with uniform digital capability or uniform relevance of GenAI use. Instead, they described variation by role, age, prior exposure, and perceived applicability to daily work. One participant explained:

*Our early AI tutorials did not work well because we treated 25-year-old doctors and 40-year-old administrators as if they needed the same thing. The doctors’ completion rate was much higher. The administrators often did not see why they should use it or how it related to their work*.

[BJ-HR03]

This quotation illustrated that uneven readiness was not simply a matter of technical ability. Participants also emphasized differences in perceived usefulness. Staff were more receptive when they could see a direct connection between GenAI use and their tasks. When relevance was unclear, completion and engagement were lower. Another participant deepened this point:

*Some, especially younger staff, want to start using it immediately and see it as a major efficiency tool. But some more senior staff still see it as something for the IT department rather than their own unit, or they feel it is too abstract. They worry that if AI generates something inaccurate and they fail to notice it, they will still be held responsible*.

[BJ-HR07]

Descriptively, participants from Sichuan more often emphasized resource and infrastructure limitations, whereas participants from Beijing more often discussed differential uptake and role-based variation in training response. However, because the study was not designed for formal site comparison and the sample size was small, these observations should be interpreted cautiously as descriptive tendencies rather than robust regional contrasts. Table 4 provides a restrained descriptive cross-site summary.

**Table 4.** Descriptive site-level patterns in hospital human resource managers’ emphasis on generative AI readiness at tertiary hospitals in Beijing and Sichuan, China, January to February 2025^a^.

Analytic area	Beijing participants more often emphasized	Sichuan participants more often emphasized
Nature of current GenAI^b^ environment	Uneven uptake, informal experimentation, role-based variation	Infrastructure constraints, operational feasibility, implementation conditions
Main concern	Who needs what type of preparation	Whether the hospital environment is ready for broader expectations
Preferred response	Differentiated training pathways	Phased implementation linked to system readiness

a Patterns are descriptive tendencies and should not be interpreted as formal regional comparisons.

b GenAI: generative AI.

### Theme 3: Participants Preferred Phased, Role-Specific Preparation Over Immediate Compulsory Standards

#### Overview

Participants’ preferred response to the emerging presence of GenAI was pragmatic rather than universalizing. They did not support a single competency mandate for all staff at the current stage. Instead, they favored phased preparation tailored to role, workflow, and organizational maturity.

#### Subtheme 3.1: Skill Development Should Be Tiered by Role and Task

Participants repeatedly argued that “AI skills” could not be treated as a single undifferentiated category. In their accounts, staff needed different forms and levels of preparation depending on the kinds of work they performed. One participant said:

*AI skills should be tiered. Some staff only need basic data and digital literacy. Others, especially those already working with AI-supported systems, need to know how to write prompts clearly and how to check whether the output is reliable. They do not need to build models, but they do need to use the tools properly*.

[SC-HR07]

It also showed that participants were not calling for advanced technical specialization for all staff. Rather, they were proposing bounded, practical, and role-linked learning.

#### Subtheme 3.2: Workflow Fit and Collaborative Design Were Viewed as Conditions for Acceptance

Participants indicated that staff acceptance depended not only on training but also on whether GenAI tools aligned with actual workflows and whether end users had a role in shaping implementation. Participants were skeptical of purely top-down rollouts in which technologies were introduced without attention to day-to-day practice. One participant described a patient education example:

*Some nurses and physicians worked together on AI tools for patient education. They could identify what patients usually ask, what language is appropriate, and where human review is still needed. They saw things that the IT staff alone could not see, and the tool became much more acceptable after that*.

[SC-HR03]

This account added important detail to the theme. The collaboration described by participants was not about building models. It concerned practical contributions to content boundaries, tone, accuracy review, and workflow integration. In that sense, collaborative design was framed as a social and organizational condition for successful GenAI use. This was reinforced by another participant from Beijing:

*We discussed this informally with clinicians, for example whether clinical departments could clearly define which routine inquiries AI could handle, which would require prior review, and which must be transferred to a human. Those rules have not been established yet. Without them, not only clinicians, but even we as managers would not feel comfortable handing communication over to AI*.

[BJ-HR01]

**Figure 2.**
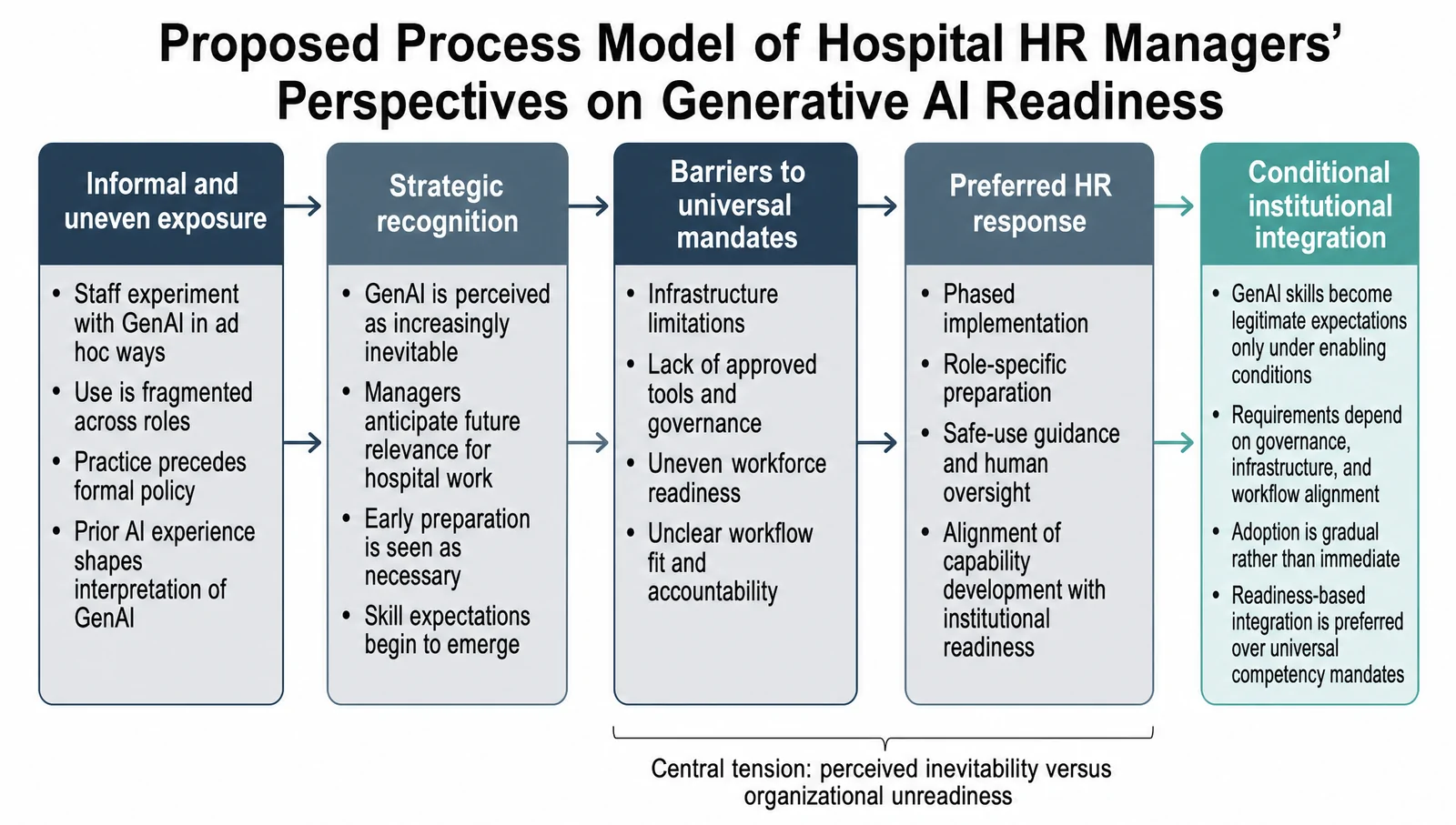
Proposed process model of hospital human resource managers’ perspectives on organizational readiness for generative AI skills, derived from semistructured interviews in a descriptive qualitative study at 2 tertiary hospitals in Beijing and Sichuan, China, January 15 to February 9, 2025. The model summarizes a pathway from informal exposure through strategic recognition and implementation barriers to a preferred phased response and conditional institutional integration; it is an empirically informed summary rather than an implementation algorithm. GenAI: generative AI; HR: human resource.

Overall, the findings indicated that hospital HR managers did not describe mature GenAI competency systems already operating within their institutions. Instead, they described an early-stage environment in which GenAI was seen as increasingly important but not yet governable through universal competency mandates. Their accounts emphasized that current use was often informal, uneven, and insufficiently guided by clear institutional rules. Figure 2 summarizes the process implied by participants’ accounts across the 3 themes.

Across both sites, participants favored a phased response anchored in infrastructure, governance, role differentiation, and workflow fit. In this sense, the study’s central contribution is not the identification of a fully developed competency framework, but the description of a preimplementation landscape in which HR managers are trying to reconcile the perceived inevitability of GenAI with the organizational conditions needed for safe, fair, and meaningful adoption.

## Discussion

### Principal Findings

This study explored how hospital HR managers perceived the emerging expectation that hospital staff should develop skills related to GenAI. Participants did not describe mature, formalized GenAI competency systems operating in their hospitals. Instead, they described an early-stage environment characterized by strategic recognition of GenAI, informal and uneven use, uncertain governance, and substantial differences in readiness across roles and organizational conditions. This pattern is broadly consistent with current health care literature showing that AI and GenAI adoption often advances faster than institutional standardization, governance, and workforce preparation.

The central tension in the findings is that GenAI is seen as increasingly inevitable, yet not presently governable through universal competency mandates. Participants did not reject GenAI as irrelevant or undesirable. Rather, they questioned whether current infrastructure, approved tools, policy clarity, workflow integration, and workforce preparedness were sufficient to justify broad formal requirements. This extends current implementation literature showing that readiness for AI in health care is multidimensional and depends on organizational and sociotechnical conditions rather than technical promise alone.

### GenAI Readiness Is Not the Same as General AI Readiness

A key interpretive issue in this study is the distinction between GenAI and broader forms of AI. Participants sometimes referred to imaging systems, diagnostics, or predictive tools when discussing preparedness. In our interpretation, such references do not function as direct evidence of GenAI use. Instead, they show that managers often make sense of GenAI through earlier institutional encounters with AI more broadly. That observation is important because it suggests that, in hospitals, GenAI is frequently understood as part of an evolving AI landscape rather than as a wholly separate phenomenon.

At the same time, preserving the distinction is analytically necessary. GenAI introduces risks and capability demands that differ from those associated with imaging, prediction, or classification systems, including hallucinated text, prompt sensitivity, variable output quality, opaque reasoning, and heightened privacy concerns related to prompt inputs [3,5]. GenAI-focused health care literature has therefore emphasized the need for new competencies related to verification, boundary awareness, human oversight, and safe operational use [4,8,9]. Our findings support that distinction at the managerial level: participants often recognized AI as a broad strategic direction, but they were still working out what GenAI specifically demands from staff and institutions [13,14].

### From Inevitability to Conditional Adoption

One of the strongest patterns in the data is that participants did not frame GenAI as optional in the long term. That perspective aligns with broader commentary suggesting that AI, and increasingly GenAI, is likely to reshape health care work, education, and administration [2,11,41]. However, the managerial response observed here is conditional rather than triumphalist. Participants did not move from “GenAI is coming” to “all staff must now be competent.” Instead, they moved from “GenAI is coming” to “we are not yet organizationally ready to require it universally.”

This matters conceptually because it complicates linear models of technology adoption. Evidence from systematic reviews shows that health care professionals’ perceptions of AI vary widely according to trust, comprehension, workflow burden, and professional concerns [13-15]. Survey work among health care workers also suggests that adoption and satisfaction are shaped by paradoxical tensions and change perceptions rather than by simple acceptance [35,36]. Studies applying Unified Theory of Acceptance and Use of Technology to GenAI use further underscore the importance of performance expectancy, facilitating conditions, and social influences. In that sense, our findings suggest that perceived inevitability can coexist with low confidence in facilitating conditions and institutional support [19,20].

### Infrastructure, Governance, and the Limits of Mandates

Participants’ concern about infrastructure should not be read narrowly as a complaint about hardware. Their accounts indicate a broader understanding of readiness that includes approved access pathways, secure systems, policy clarity, workflow integration, and accountability structures. This is consistent with recent health care GenAI literature emphasizing secure infrastructure, data governance, privacy protection, and institutional oversight [23,25,42]. Work on responsible AI in health care similarly identifies transparency, fairness, human-centeredness, and accountability as essential implementation principles [43,44].

GenAI is also distinctive because it can enter organizations informally through public tools before enterprise systems and governance are in place. Preliminary evidence already shows active experimentation with GenAI across health care domains [1,6]. Yet policy and implementation commentaries consistently warn that responsible use requires safeguards, regulation, and institutional governance [24,25,45]. The present findings suggest that HR managers are already encountering this mismatch between ease of access and institutional governability.

This issue is especially relevant in China, where AI in health care is advancing rapidly but remains shaped by evolving regulatory, privacy, and evaluation frameworks [37-39]. Recent analyses of Chinese medical AI regulation underscore the complexity of supervising dynamic algorithms, multimodal systems, and software-based tools [38-40]. Our findings do not test regulatory implementation directly, but they are consistent with a context in which governance maturity may lag behind strategic enthusiasm.

### Role-Specific Preparation Rather Than Uniform Competency Models

Another important finding is participants’ rejection of a single undifferentiated expectation across staff groups. They repeatedly described readiness as varying by role, prior exposure, confidence, and perceived relevance to daily work. This supports existing competency literature showing that health care AI capability should not be treated as a single universal construct [9,10]. Educational commentary in medicine has likewise argued for targeted rather than generic AI training [9,11,12]. Some recent work even proposes new physician roles and tiered competency trajectories for clinically integrated AI environments [9,46].

Our findings extend that logic beyond clinicians by bringing in the HR-management perspective. Participants were not merely saying that “training is needed.” They were indicating that premature standardization could be ineffective and inequitable because staff groups differ in role relevance, prior digital exposure, and ability to evaluate outputs safely. Evidence from studies of health care workers’ knowledge, attitudes, and change readiness supports the view that readiness is heterogeneous rather than uniform [35,47]. For some roles, basic safe-use literacy may be sufficient. For others, especially communication-facing, documentation-heavy, or implementation-facing roles, more advanced preparation may involve prompting, review of generated outputs, and governance awareness. That inference is directly compatible with GenAI-focused capability arguments in the current literature [5,9].

The HR literature also supports this role-differentiated interpretation. Research on algorithmic technologies in HRM describes AI as reshaping not only processes but also skill demands, job design, governance, and employee relations [28,48,49]. Work on GenAI in HRM further highlights the need to define human versus machine roles carefully and to manage risks alongside efficiency claims [32,34]. In that sense, participants’ emphasis on phased, role-specific preparation is not only an operational preference. It is a plausible response to the broader problem of aligning AI capability expectations with actual work.

### Workflow Fit and Collaborative Sociotechnical Design

Participants also emphasized that staff acceptance depends on whether GenAI fits real work. This is strongly aligned with the sociotechnical implementation literature, which shows that AI success depends on alignment with clinical or administrative workflow rather than on technical capacity alone [17,18]. Recent studies of ambient AI scribes provide a useful parallel. Clinicians report benefits such as reduced documentation burden and perceived efficiency gains, but also identify customization, usability, trust, and workflow integration as central to acceptance [50-52]. Additional qualitative and comparative work on ambient AI documentation similarly shows that implementation outcomes vary across systems and depend on the details of use and local context [53,54].

The subtheme concerning collaborative workflow design is therefore more than a minor implementation preference. Participants suggested that GenAI-related tools become more acceptable when end users help define use cases, limits, language, review processes, and handoff points to humans. This is consistent with evidence that physician input can improve GenAI performance in clinically relevant settings and that human-AI interaction remains central to reliable deployment [55]. It is also consistent with a recent governance commentary that argues frontline professionals, including nurses, should be visible participants in AI oversight and implementation [42].

### Theoretical Implications

The findings have 3 main theoretical implications. First, they support a shift from strong competency-framework language toward readiness and preimplementation language when organizations remain in an uneven and only partially governed phase of GenAI uptake. This is more consistent with current health care evidence showing adoption alongside substantial barriers, rather than widespread institutional maturity [17,19,20].

Second, the findings suggest that organizational perceptions of GenAI are mediated by legacy encounters with other AI technologies. GenAI adoption in hospitals may therefore be layered onto preexisting institutional understandings of AI, trust, and digital modernization. That helps explain why participants moved between GenAI and broader AI examples when discussing preparedness.

Third, the study complicates simple acceptance narratives by showing that strategic importance and organizational unreadiness can coexist. This duality is consistent with health care literature showing that AI is often simultaneously promising, contested, and dependent on governance and trust [13,15,56,57]. Public studies of AI in health care also show that trust shapes expectations and comfort, further underscoring that readiness is not merely technical [56,57].

### Practical Implications

For hospital HR departments, the findings suggest that GenAI capability development should begin with readiness assessment rather than immediate standard setting. A practical starting point is to identify where GenAI is already being used informally, which roles perceive it as relevant, what concerns are already visible, and where secure institutional alternatives are absent. This sequencing is consistent with health system adoption surveys and hospital implementation frameworks [18,20].

For hospital leadership, the data imply that capability development and infrastructure development should proceed together. Secure access, data-handling rules, verification expectations, governance structures, and escalation pathways are not optional extras. They are preconditions for sustainable implementation. Recent GenAI scholarship in health care and policy makes the same point [23-25].

For policymakers and professional bodies, the findings suggest that early guidance may be more useful if framed around minimum safe-use capabilities, privacy-aware use, role-based progression, and governance responsibility rather than universal competency mandates. This is compatible with emerging reporting and governance frameworks for GenAI-driven health tools [7,8,42]. It is also compatible with calls for responsible AI regulation and clearer federal or system-level guardrails [24,25].

It is also worth noting that the findings should be interpreted within the setting of Chinese tertiary hospitals. China has seen rapid expansion of health care AI applications and commercialization, alongside ongoing evolution in regulation and infrastructure. Our data suggest that even in tertiary settings, GenAI-related expectations may emerge before institutional conditions are fully standardized. That pattern is likely not unique to China, but the Chinese context makes issues of governance, digital variation, and implementation timing especially salient.

### Limitations

This study has several limitations. It was conducted in 2 tertiary hospitals and therefore does not represent all Chinese hospitals or international health care systems. It focused on HR managers, not clinicians, IT leaders, or senior administrators. Because the interviews addressed an emerging topic, participants sometimes moved between GenAI and broader AI examples; analytically, we treated non-GenAI references as evidence of prior AI exposure rather than as direct evidence of GenAI practice. Finally, the quotations were translated from Chinese into English, and although they were edited for readability, some nuance in tone or colloquial meaning may have been attenuated. Caution about interpreting attitudes and expectations in AI studies is consistent with the broader literature on health care professionals’ experience, trust, and concern.

### Future Research

Future research should proceed in several directions. Multistakeholder studies should compare HR managers’ views with those of clinicians, IT teams, executives, and frontline staff. Comparative work across hospitals with different levels of digital maturity could clarify how infrastructure and governance shape GenAI readiness. Longitudinal studies should examine whether perceptions change once secure enterprise GenAI systems and formal policies are introduced. Instrument-development work could also build on current findings to create practical readiness-assessment tools for hospital GenAI implementation. Each of these directions is supported by the current state of the literature, which shows rapid growth in AI use alongside ongoing uncertainty about governance, trust, implementation, and workforce preparedness.

### Conclusions

Hospital HR managers describe GenAI not as a fully institutionalized competency domain, but as an emerging and uneven organizational reality. They recognize GenAI as increasingly relevant to hospital work, yet they do not view current conditions as sufficient to support universal competency mandates. Instead, they frame readiness as conditional on infrastructure, secure access, governance, workflow fit, and role-specific preparation. Their accounts suggest that hospitals are not simply deciding whether to adopt GenAI; they are negotiating when, for whom, and under what safeguards GenAI-related skills become legitimate expectations. This interpretation is consistent with current literature on early GenAI adoption in health care, which emphasizes both increasing use and substantial implementation barriers.

## Supplementary material

10.2196/74339 Multimedia Appendix 1 Interview guide (English translation).

## Abbreviations

GenAI: generative AI
HR: human resource
HRM: human resource management
