# Supplementary material for: Hospital Human Resource Managers’ Perspectives on Organizational Readiness for Generative AI Skills: Qualitative Descriptive Study
Source: JMIR Med Inform. 2026 Sep 17;14:e74339. doi: 10.2196/74339 (PMC13585017; doi:10.2196/74339)
Supplement: Supplementary file 1 [file medinform-v14-e74339-s001.pdf]

Appendix 1: Interview guide (English translation)

1. Please describe your current HR role, main responsibilities, and which staff groups you oversee.

2. How long have you worked in hospital HR management, and what prior roles shaped your perspective on workforce development?

3. In your hospital, who makes decisions about adopting GenAI-related tools, and how is HR involved?

4. What GenAI-related use is currently happening in your hospital (formal programs, informal use, or none)?

5. When you hear "GenAI competency," what does it mean in your hospital context?

6. Which GenAI competencies should be expected of all staff, if any, and which should be role-specific?

7. Do you think GenAI competency should become a formal, required competency standard in hospitals? Why or why not?

8. What benefits do you anticipate from developing GenAI competency (for operations, workforce development, or patient-related work)?

9. What risks or concerns do you see (eg, safety, privacy, bias, accountability, misinformation, overreliance), and which matter most?

10. What barriers would make a competency requirement difficult to implement in your hospital (eg, infrastructure, policies, tool access, time, incentives)?

11. How does readiness vary across roles or cohorts (eg, clinicians vs administrators, younger vs older staff), and why?

12. If you were designing a competency pathway, what tiers or stages would you propose for

different roles?

13. How should GenAI competency be taught and supported in practice (formats, resources, incentives, protected time)?

14. How should competency be assessed and maintained over time, and what would “minimum acceptable competency” look like?

15. What governance and participation are needed to implement this responsibly (approved tools, acceptable use rules, incident response, frontline involvement), and what are your top recommendations for the next 3 to 6 months?
